# A biological plausible recurrent model of V1 hypercolumns

**DOI:** 10.1186/1471-2202-12-S1-P48

**Published:** 2011-07-18

**Authors:** Atahan Afşar, Tunca Ulubilge, Baran Çürüklü

**Affiliations:** 1 Faculty of Engineering and Natural Sciences, Sabanci University, Istanbul, 34956, Turkey; 2 School of Innovation, Design and Engineering, Mälardalen University, Västerås, 721 23, Sweden

## 

A biological plausible model of hypercolumn of V1 layer in the Primary Visual Cortex, modeled in the NEST Environment [1], is presented. The model addresses experimental findings on emergence of orientation selectivity which occurs in the V1 [2]. The network model is derived from the Bayesian confidence propagation neural network, which was presented earlier [3,4]. It is hypothesized that a modular recurrent network model can be used to address orientation selectivity mechanism [2]. Thus, the columnar organization of primary visual cortex is assumed [5]. The network consists of 16 minicolumn models each representing an orientation, ranging from 0^o^ to 168.75^o^, with the angular distance of 11.25^o^ between two adjacent minicolumn models. LGN input is broadly tuned, half-width of half-height (HWHH) is 40 ^o^. Excitatory->Excitatory network targets all neurons with the probability of 60% inside the host minicolumn with a HWHH of 25^o^ as a function of distance (ESPSs = 3.15 mV). Inhibitory->Excitatory is connected with the probability of 40% (ISPSs = -5.85mV). Excitatory->Inhibitory connections target all neurons with the probability of 40%, and HWHH of 67.5^o^ as a function of distance (ESPSs = 1.35 mV). Furthermore, LGN input is 1/3 of cortical excitation. Hypercolumn model also reflects biological phenomenon of background activity caused by random cortical inputs, as suggested by the experimental findings. In the absence of LGN input, background activity of the population is around 0.5-2 spikes/sec.

## Conclusions

**Figure 1:**
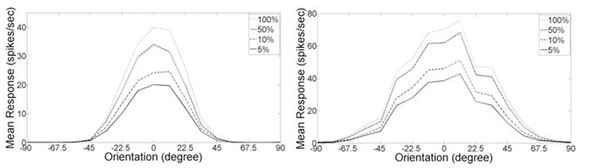
Mean excitatory population activity. A) Orientation response curves of a cortical hypercolumns. LGN input and cortical excitatory and inhibitory connections are present B) Response to broadly tuned LGN input in absence of cortical excitatory and inhibitory connections.

LGN input ranges from low to high contrast (5%, 10%, 50%, and 100%), and is fed into the neurons during 2 seconds for each contrast level (mean activities of the excitatory population is shown in Figs 1A and 1B). Simulation results suggest that cortical connections of excitatory and inhibitory neurons play an important role in sharpen of the broadly tuned LGN input. Emergence of contrast invariance of orientation selectivity is also evident (Fig. 1A.). As demonstrated in this specific simulation the cortical network is also efficient in correcting network activity, which is the function of the LGN input solely; when cortical network is absent minicolumn model representing 15 ^o^ is most active, whereas in presence of cortical connections 0 ^o^ comes out as the winner.
